# Supplementary material for: Severe Acute Myocarditis after the Third (Booster) Dose of mRNA COVID-19 Vaccination
Source: Vaccines (Basel). 2022 Apr 8;10(4):575. doi: 10.3390/vaccines10040575 (PMC9024648; doi:10.3390/vaccines10040575)

Supplementary Appendix

Fulminant Myocarditis After BNT162b2 Vaccine Booster Against COVID-19

Asleh R et al.

Figure S1. Full 2-lead recording from a wearable cardioverter-defibrillation (LifeVest) of the same event presented in figure 2S, demonstrating sustained monomorphic ventricular tachycardia lasting for 94 seconds, ultimately terminated by a successful electrical shock.

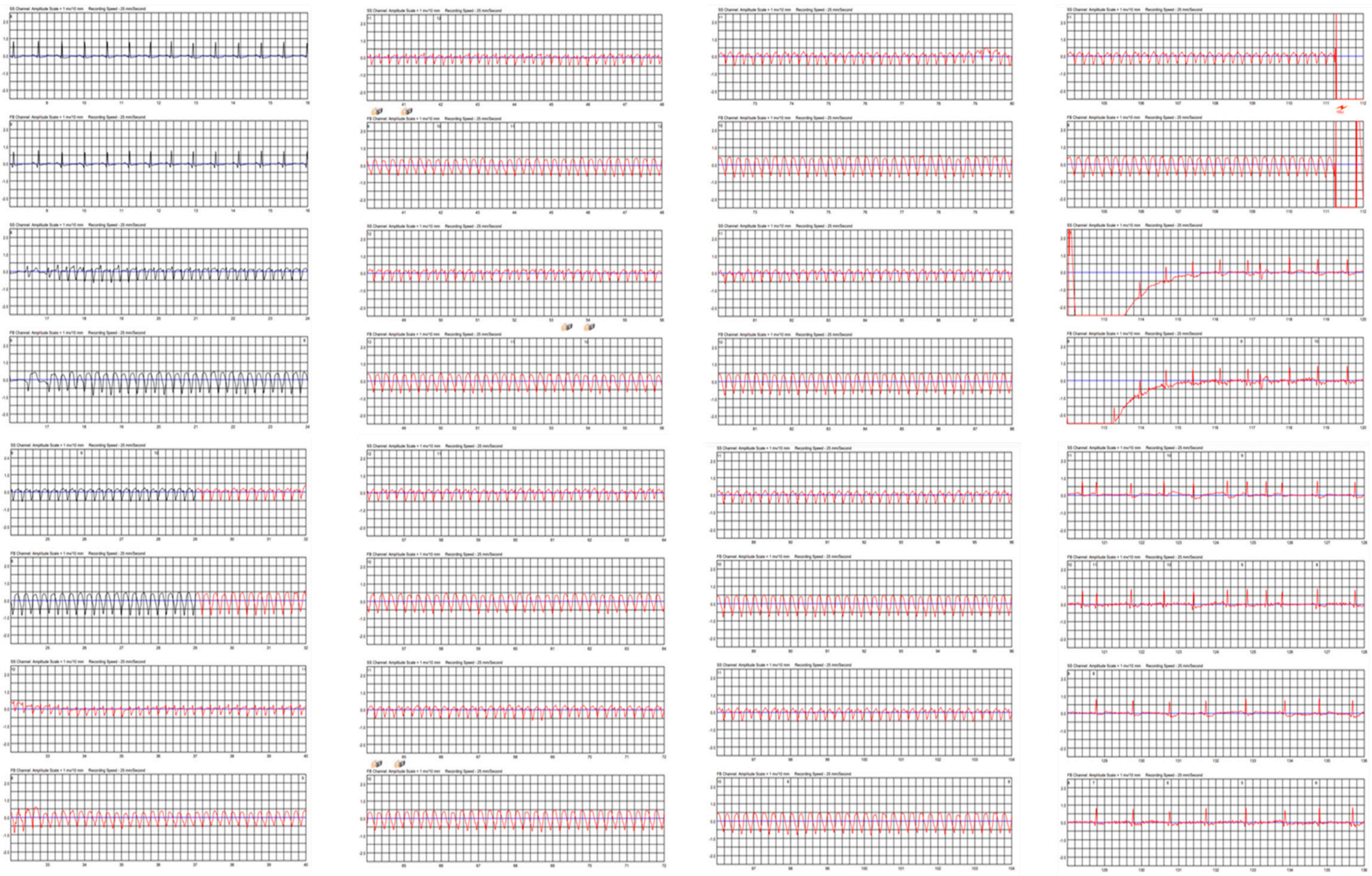

Figure S2. Cardiac MRI (3T) at 3 months of follow-up. (D) A short axis view LGE image showing a transmural LGE in the inferolateral wall (red arrow). (E) A T2 mapping image showing no myocardial edema with normal regional T2 values (up to 45 milliseconds) in the lateral and inferolateral walls. (F) A three-chamber view LGE image showing transmural LGE on a thin and aneurysmatic inferolateral wall segment (red arrow).

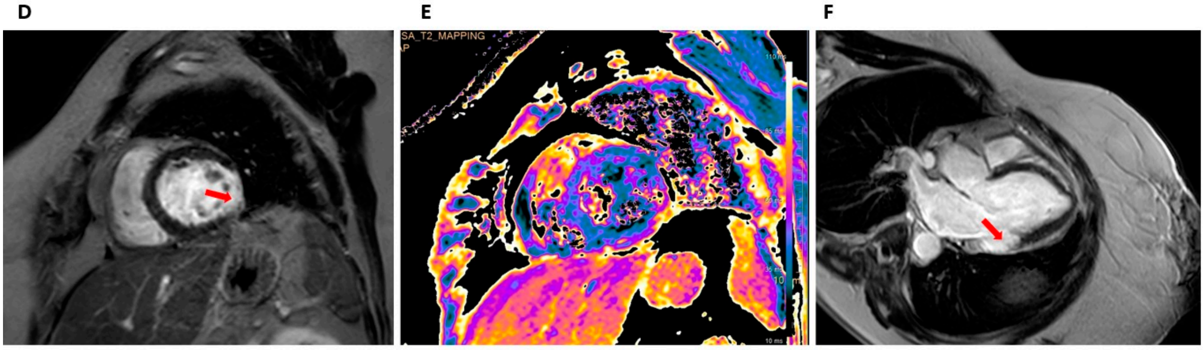

Figure S3. Additional histological findings from an endomyocardial biopsy performed after 3 months of follow-up following the index hospitalization. Hematoxylin-eosin stains of heart-tissue specimens showed resolution of myocarditis without inflammation, cardiomyocyte damage, or interstitial edema (Panel A). Masson`s trichrome staining for collagen deposition highlighted interstitial fibrosis within the myocardium (blue dye) (Panel B). Immunohistochemistry stains showed no lymphocyte (Panel C, CD3 immunostaining for T-cells, and Panel D, CD20 stain for B-cells) or macrophage (Panel E, CD68 stain) infiltration. Original magnification, x200; scale bars, 100 µm.

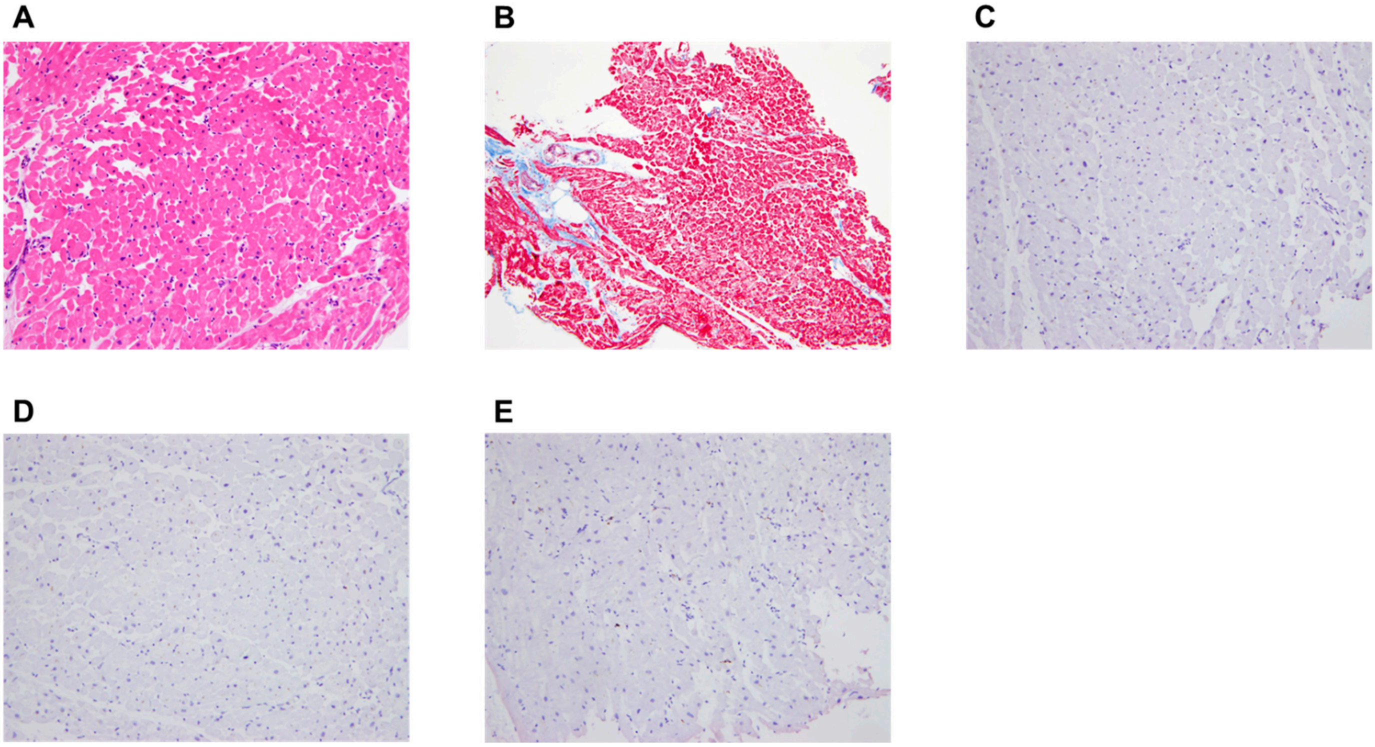

Supplement: Supplementary file 1 [file vaccines-10-00575-s001.zip › vaccines-1649235-supplementary.pdf]
